# Lung long distance: histopathological changes in lung tissue after COVID-19 pneumonia

**DOI:** 10.3325/cmj.2024.65.501

**Published:** 2024-12

**Authors:** Grgur Salai, Jasna Tekavec-Trkanjec, Ivona Kovačević, Čedna Tomasović-Lončarić, Arijana Pačić, Mirna Vergles, Divo Ljubičić, Daria Cvetković-Kučić, Ivica Lukšić, Bruno Baršić

**Affiliations:** 1 Department of Pulmonology, University Hospital Dubrava, Zagreb, Croatia; 2 Clinical Department of Pathology and Cytology, University Hospital Dubrava, Zagreb, Croatia; 3 Clinical Department of Diagnostic and Interventional Radiology, University Hospital Dubrava, Zagreb, Croatia; 4 School of Medicine, University of Zagreb, Zagreb, Croatia; 5 University Hospital Dubrava, Zagreb, Croatia; 6 Infectious Disease Service, University Hospital Dubrava, Zagreb, Croatia; The first two authors contributed equally.; Salai et al: Histopathological changes in lung tissue after COVID-19 pneumonia

## Abstract

**Aim:**

To investigate histopathological changes in the lung tissue of long-COVID patients.

**Methods:**

In this cross-sectional study, transbronchial lung biopsy was performed in long-COVID patients with persisting symptoms and radiological abnormalities. Histopathologic analyses were performed by using hematoxylin-eosin, Martius, Scarlet and Blue, Movat's, thyroid transcription factor 1, CD34, and CD68 staining.

**Results:**

Adequate biopsy samples were obtained from 29/32 patients. The median (Q1-Q3) time from disease onset to biopsy was 13 (9-20) weeks. We observed several histopathologic patterns: DAD with vascular abnormalities (VA) (n = 8); VA with inflammatory pattern (n = 4); inflammatory pattern (n = 13), and fibrotic pattern (n = 4). VA included capillary thrombi, dilated venules, and dissection of small pulmonary arteries. DAD with VA was detected up to the 9th week from the onset of disease; inflammatory pattern from the 8th to 28th week (4 patients with this pattern biopsied in the 11th-13th week had accompanying VA); and a predominantly fibrotic pattern was found at weeks 8, 10, 48, and 49.

**Conclusion:**

Our study observed a slow recovery of lung tissue with long-lasting DAD and VA, likely followed by interstitial inflammation or focal fibrosis. These findings might be the underlying cause of the slow recovery of long-COVID patients.

Long COVID is defined as an infection-associated chronic condition that occurs after SARS-CoV-2 infection and is present for at least three months as a continuous, relapsing and remitting, or progressive disease state that affects one or more organ systems (1). This definition was endorsed by the US Centers for Disease Control and Prevention in July 2024 (2). However, during the pandemic, acute and long COVID were commonly differentiated at 4 weeks from the onset of infection (3-6). This differentiation was also included in the NICE guidelines published at the time (7,8). The updated NICE guidelines further divided long COVID into “ongoing symptomatic COVID” (4-12 weeks) and “post-COVID” (>12 weeks) (8).

Pathophysiological mechanisms of long COVID are not understood, are likely multifactorial, and involve chronic inflammation and immune status alteration (5). The most common respiratory symptoms in long-COVID patients are dyspnea and cough (9), with the most common pulmonary function test abnormality being a reduction in carbon monoxide diffusion capacity. The most frequently reported radiological features of long COVID are low resolution of ground glass opacities and fibrotic-like changes (10).

Histopathological studies focused on autopsy-based analyses of patients with severe acute COVID-19 most commonly described diffuse alveolar damage (DAD) (11,12). Vascular changes, including thrombosis, endothelialitis, and angiogenesis have also been observed (12). Histopathological reports in acute COVID-19 most frequently included the presence of hyaline membranes, involvement of endothelial and interstitial cells, alveolar hemorrhage, and microthrombi and intraalveolar fibrin deposits (13).

Ravaglia et al categorized the findings of 10 long-COVID patients who underwent lung cryobiopsy based on the histopathological pattern. Three clusters emerged, as follows: the “chronic fibrosing” cluster, characterized by a progression of likely pre-existing interstitial pneumonias; the “acute/subacute injury” cluster characterized by different grades of response to lung injury; and the “vascular changes” cluster (including dilation and distortion of vascular venules and capillaries) (14). In the setting of long COVID, important findings were obtained on lungs explanted from patients undergoing lung transplantation (15): In the explanted lungs of three patients (weeks 9-14), Bharat et al found extensive interstitial thickening, fibrosis, bronchiolitis, bronchiolar fibrosis, honeycombing, as well as alveolar hemorrhage and evidence of iron deposition in the alveolar macrophages (15,16). Aesif et al reported on the findings in the explanted lungs of a 57-year old male who underwent lung transplantation at about 11 weeks post-COVID. They observed mild, diffuse interstitial chronic inflammation with diffuse, relatively uniform-appearing interstitial expansion, a pattern vaguely resembling non-specific interstitial pneumonia (NSIP). This pattern was accompanied by numerous hemosiderin-laden and foamy macrophages within airspaces, as well as foci of honeycombing (15,17). Interestingly, Kehara et al observed chest CT improvement of the native lung in 5/13 single-lung transplant recipients at follow-up (18). All the explanted lungs of these patients had advanced interstitial lung fibrosis and extensive lung injuries on histological examination (18), indicating the reversibility potential (19). Namely, the reversal of fibrosis-associated lesions, mainly on chest imaging in the setting of (long) COVID, has been observed and is a promising avenue to be explored (20,21).

Even though many studies focused on radiological changes and pulmonary function testing in long-COVID patients suffering from respiratory symptoms, histopathological changes obtained by bronchoscopy have rarely been researched. We aimed to investigate lung-tissue changes affecting long-COVID patients (from 4 weeks after disease onset). Therefore, we conducted a cross-sectional histopathological study of long-COVID patients suffering from persistent respiratory symptoms after having been diagnosed with moderate to severe COVID-19 pneumonia.

## Patients and methods

This cross-sectional study of long-COVID patients was approved by the Institutional Ethics Committee of Dubrava University Hospital. All participants signed an informed consent form. The study conformed with the Declaration of Helsinki. Bronchoscopies were performed from 2021 to June 2023. Long COVID was defined at the 4-week census, as per the NICE guidelines relevant at the time (7).

Inclusion criteria were a) age older than 18 years, b) history of COVID-19 pneumonia confirmed by polymerase chain reaction (PCR) testing, c) respiratory symptoms persisting for at least 4 weeks after COVID-19 onset, d) at least 20% of lung parenchyma affected as visible on high-resolution chest computerized tomography (HRCT) at the time of pre-bronchoscopy assessment.

Exclusion criteria were previously known interstitial lung or autoimmune disease, previously known lung malignancy, or a positive nasopharyngeal swab PCR test for SARS-CoV-2 at the time of bronchoscopy. Positive PCR test as an exclusion criterion was a requirement of the Institutional Ethics Committee as, at the time of study initiation (2021), bronchoscopies could not be performed in out-patients who were SARS-CoV-2 positive.

HRCT scans were analyzed by a board-certified radiologist, specialized in thoracic radiology. Bronchoscopy with transbronchial (forceps) lung biopsy with at least 5 biopsies was performed for each participant. Samples were then fixed in formalin solution and stained with hematoxylin and eosin, and additionally with Martius, Scarlet and Blue; Movat's; thyroid transcription factor 1; CD34; and CD68 staining.

Histopathological samples were analyzed independently by two board-certified pathologists specialized in lung tissue analysis. In case of diagnosis mismatch, an agreement was reached by consensus. Samples were deemed adequate if more than 20 alveoli were identified (22). All cases were presented and discussed at regular multidisciplinary team meetings.

Participants' data were collected retrospectively from their medical history. Comorbidities known at the time of pre-bronchoscopy evaluation were summarized by employing the Charlson comorbidity index. Parametric variables are reported as means ± standard errors from the mean (SE). Non-parametric variables are reported as medians (1st and 3rd quartiles, Q1-Q3). Participants' survival was assessed at a single time point, at 18 months from COVID-19 diagnosis by performing a telephone call. No participant was lost to follow-up.

Histopathological findings were grouped into categories based on major patterns and plotted on a timeline in weeks, which was depicted using Draw.io. For the purpose of presenting patient characteristics, histological patterns were further divided into four groups. The four groups were compared for each parameter by using a one-way non-parametric analysis of variance (Kruskal-Wallis test). Dwass-Steel-Critchflow-Flinger pairwise comparisons were performed, but due to redundancy and lack of relevance, they were not reported.

The predominantly inflammatory/fibrotic patterns pertain to the histopathological findings that could not be classified into a distinct disease entity (eg, one of the idiopathic interstitial pneumonias) or according to histopathology, after a multidisciplinary discussion and integration with clinical and radiological findings.

## Results

Our study enrolled 32 participants (3 women). In 31 patients, transbronchial biopsy was performed at a single time point, and in one patient, three bronchoscopies with transbronchial biopsy were performed due to clinical indication; therefore, 34 bronchoscopies were performed. Participants' median age at bronchoscopy was 65 (54.5-71.3) years. Patients' general characteristics are presented in Table 1.

**Table 1:** Participants' general characteristics

Participants (N)	N = 32
Age at the time of bronchoscopy; median (Q1-Q3)	65 (54.5-71.3) years
Sex; n (%)	
male	29 (90.6)
female	3 (9.4)
Charlson comorbidity index; median (Q1-Q3)	2 (1.75-4)
High-resolution computerized tomography, % of lung involvement; median (mean ± standard error)	44.5% (±3.1)
Alive at 18 months of follow-up; n (%)	28 (87.5)

Out of 34 performed bronchoscopies, 3 (8.8%) biopsy samples were inadequate for histopathological characterization. No serious adverse events were reported. Bronchoscopies were performed at a median of 13 (9-20) weeks after the onset of COVID-19 symptoms.

We observed several histopathological patterns: a) DAD, b) predominantly inflammatory pattern of the interstitium (which could not be classified into a known histopathological entity), c) predominantly fibrotic pattern (which could not be classified into a known entity), d) fibrosing nonspecific idiopathic pneumonia (NSIP), e) organizing pneumonia (OP), f) vascular abnormalities (including intracapillary thrombi, dilation of postcapillary venules, pulmonary arteriole dissection), and g) other findings (pulmonary ossification, lepidic adenocarcinoma).

**Figure 1:**
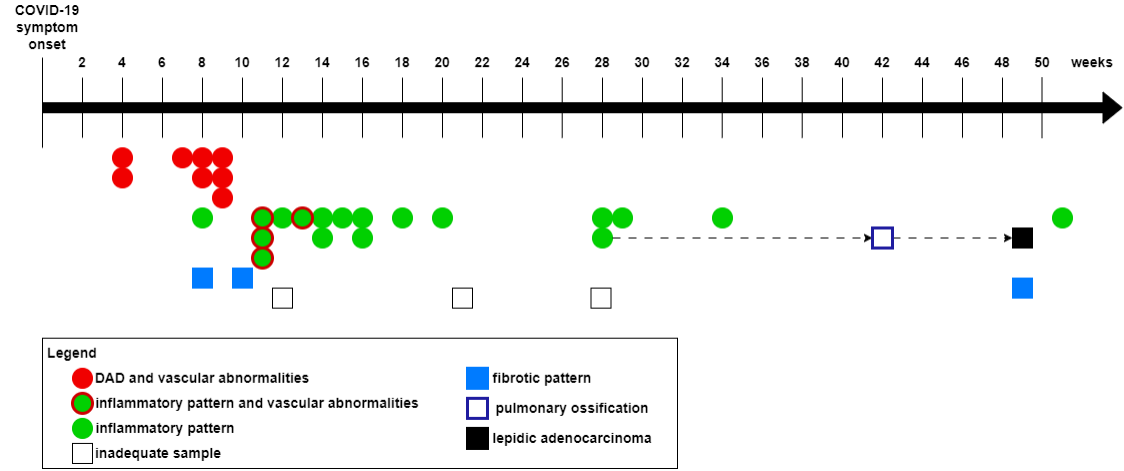
Histopathological findings plotted on a timeline in weeks from COVID-19 symptom onset to the time of bronchoscopy. Diffuse alveolar damage (DAD) with vascular abnormalities was present only in the early stage of long COVID (weeks 4-9). During the later long-COVID period (weeks 8-29), inflammatory pattern was common. Biopsies performed in the 11th and 13th week revealed an inflammatory pattern with vascular abnormalities. Several other histopathological patterns were also found: pulmonary ossification and lepidic adenocarcinoma. For clarity purposes, organizing pneumonia (biopsied at weeks 20 and 34) and fibrosing non-specific interstitial pneumonia (NSIP) (biopsied at week 51) were clustered into the “inflammatory pattern” group.

**Figure 2:**
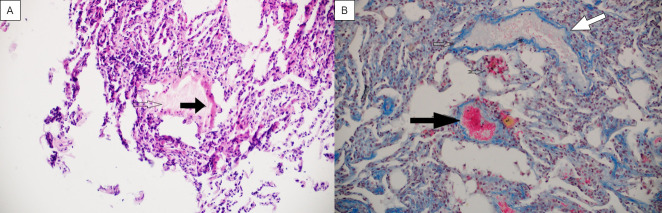
Vascular abnormalities observed under light microscope. (**A**) Diffuse alveolar damage pattern with hyperplastic type-II alveolar cells and hyaline membranes (arrow), hematoxylin eosin ×100. (**B**) Fibrin capillary thrombus (black arrow) and extremely dilated pulmonary venula (white arrow); Martius, Scarlet and Blue ×200.

**Figure 3:**
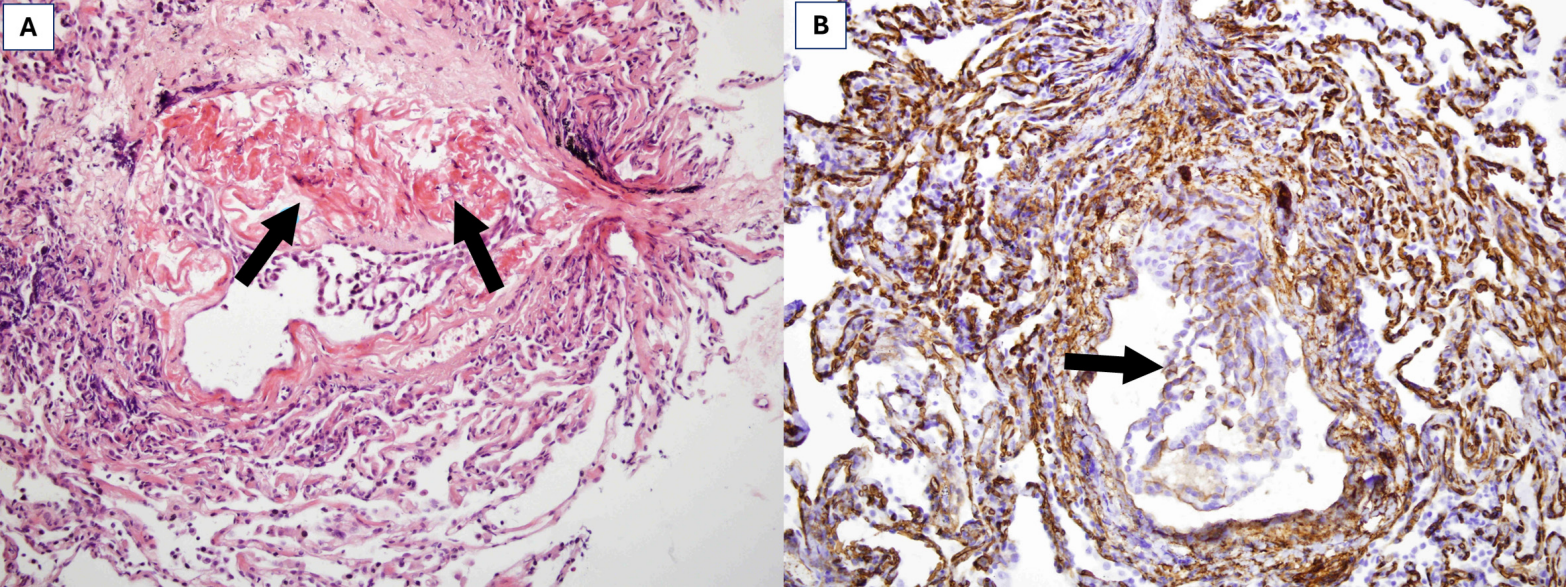
Dissection of a small pulmonary artery: (**A**) fibrin accumulation in a torn vascular wall between intima and media (arrows), hematoxylin eosin ×200. (**B**) CD34 staining showing endothelial cells sloughed into the vessel lumen, ×200.

Histopathological findings were plotted on a timeline depending on the time of biopsy (Figure 1). Eight (25%) participants biopsied during an earlier long-COVID period (weeks 4-9) had DAD with accompanying vascular abnormalities (Figure 2 and Figure 3). Fifteen (46.9%) patients had a predominantly inflammatory pattern (biopsied at weeks 8-29), of which 4 (biopsied at weeks 11-13) had associated vascular abnormalities. OP was found in 2 patients, biopsied at the 20th and 34th week. The participant biopsied at the 34th week had an accompanying finding of pulmonary ossification. A predominantly fibrotic pattern was found in 3 participants, biopsied in the 8th, 10th, and 49th week. One patient, biopsied in the 51st week had changes compatible with fibrosing NSIP, which was diagnosed at the multidisciplinary meeting. Participants' characteristics based on major histopathological groups are presented in Table 2. There were no significant differences in participants’ age, Charlson’s comorbidity index, percentage of lung involvement on HRCT, or lethal outcomes among the groups.

**Table 2:** Participants' characteristics based on major histopathological findings*

	DAD + VA	Inflammatory pattern + VA	Inflammatory pattern^†^	Fibrotic pattern	*P* value
N	8	4	14	3	
Time from disease onset in weeks; median (min - max)	7.25 (4-9)	11.5 (11-13)	16 (14-26)	10 (9-30)	<0.001
Age; median (Q1-Q3)	59.5 (53-65.3)	63.5 (54.3-72.5)	66 (58.5-76)	66 (52-67.5)	0.36
Female sex; n (%)	1 (12.5%)	0	1 (7.2)	0	0.83
Charlson comorbidity index; median (Q1-Q3)	2 (1- 3.25)	2.5 (1-4.25)	3.5 (2 -4)	2 (1-2.5)	0.4
HRCT, percentage of lung involvement median (Q1-Q3)	30 (27.5 - 42.5)	50 (37.5- 62.5)	40 (37.5-50)	65 (52.5-72.5)	0.15
Lethal outcome^‡^ n (%)	0	0	2 (14.3)	0	0.26

*Abbreviations: DAD – diffuse alveolar damage; HRCT – high resolution computerized tomography; NSIP – nonspecific interstitial pneumonia; OP – organizing pneumonia; VA – vascular abnormalities.

^†^For the purpose of patient clustering, inflammatory pattern includes patients with OP and fibrosing NSIP.

^‡^Lethal outcome assessed at 18 months from disease onset.

An 80-year-old man underwent bronchoscopy three times due to clinical indication: a bronchoscopy performed at 28 weeks revealed a predominantly inflammatory pattern; a bronchoscopy performed at 42 weeks revealed pulmonary ossification. A third bronchoscopy, performed due to progression of unilateral and regression of contralateral infiltrates, uncovered lepidic adenocarcinoma.

## Discussion

Our cross-sectional study of 32 long-COVID patients revealed several histopathological patterns: DAD with vascular abnormalities, predominantly inflammatory (with or without vascular abnormalities), and predominantly fibrotic pattern. Interestingly, only 3 women were enrolled: one had an inadequate sample and one had DAD with vascular abnormalities (biopsy at 4 weeks from disease onset). The reason for the predominance of male patients is not completely clear. However, we included patients who had had severe or moderate COVID-19 pneumonia. Evidence indicates that male sex is a potential risk factor for more severe forms of COVID-19 (23–25). Nearly all countries with known sex-disaggregated data showed a male bias in COVID-19 mortality, and the risk of death was almost 1.7 times greater in men (23).

When plotting the pathohistological results on a timeline (Figure 1), we observed an interesting trend. Namely, patients who underwent lung biopsy in the early long-COVID period (weeks 4-9) had DAD with vascular abnormalities, while those in the later stages (weeks 8-29) had a predominantly inflammatory pattern. Four patients biopsied from the 11th to 13th week had interstitial inflammation accompanied by vascular abnormalities. These findings suggest that our observations might represent a continuum of histopathological changes:

DAD with vascular abnormalities has been the most frequent finding in patients with COVID-19 acute respiratory distress syndrome (12,26). Early DAD (“exudative phase”) is characterized by intraalveolar and interstitial edema (12,27). Later stages of DAD (“organizing phase“) occur days to weeks following initial lung injury. At this time, fibroblast-rich granulation tissue can be observed in the alveolar septa. Tissue-repair mechanisms are activated: proliferation of type-II alveolar epithelial cells leads to reepithelization of the injured septa, and alveolar exudates become more cellular (12,27). Non-COVID-related DAD completely or near-completely resolves in weeks and months, with varying degrees of loss of alveolar septa and persisting fibrosis (27). Furthermore, most cases of DAD show unspecific histopathologic changes that can seldom point to an underlying process (27). Therefore, patients with a predominantly inflammatory pattern (weeks 8-29) might be in a prolonged recovery phase of DAD. This hypothesis is further strengthened with (remaining) vascular abnormalities observed in patients biopsied at weeks 11-13, which might represent such “transitional forms.” Likewise, patients with a predominantly fibrotic pattern (weeks 8, 10, 49) might have had resolution of inflammation due to abundant fibrosis as a recovery mechanism.

Vascular abnormalities were present up to 13 weeks after symptom onset. These findings shed further light on the role of severe endothelial injury, which had previously been identified during acute COVID-19. Namely, in their post-mortem analysis, Ackermann et al found that alveolar capillary microthrombi were 9 times as prevalent in patients with COVID-19 as in influenza patients. Their histopathological analysis showed widespread thrombosis with microangiopathy (28). Price et al hypothesized that the interaction of inflammation and coagulation causes lung microcirculatory thrombotic disease. Furthermore, they postulated that virus-induced pyroptosis leads to a proinflammatory cascade, which further leads to the infiltration of immune cells and establishment of a positive feedback loop mechanism. Tissue factor, which is upregulated during inflammation, causes pro-coagulation state and aids in fibrin formation, which then leads to clotting in the small vessels. Occluded microvasculature in these vessels likely contains fibrin, platelets, and neutrophils, which become confined in neutrophil extracellular traps (29), a process that ultimately results in a loss of effective gas exchange.

Therefore, taken together, long-lasting DAD and vascular abnormalities (which are likely followed by interstitial inflammation or focal fibrosis) might cause prolonged respiratory symptoms and impaired lung function tests, ie, lead to slow recovery in these patients.

Even though, at the beginning of the pandemic, acute COVID was radiologically associated with OP (30), we found only two such histopathologically confirmed cases.

A 41-year-old male patient with no comorbidities had a fibrosing NSIP, which was observed 51 weeks post COVID-19. He was initially hospitalized due to COVID-19-related bilateral pneumonia with ground glass opacities and was mechanically ventilated for 15 days. He was treated with prolonged systemic corticosteroid therapy both in hospital and after initial acute phase resolution. Subsequent HRCT revealed a fibrosing pattern concordant with fibrotic NSIP, which was diagnosed at multidisciplinary meeting. During the follow-up period, the patient showed a rapid decline in lung function and is now waiting for lung transplantation. It is impossible to exclude a preexisting asymptomatic NSIP, which might have been exacerbated by SARS-CoV-2 pneumonia.

Another interesting case was that of an 80-year-old man who underwent bronchoscopy three times. In his case, COVID-19 pneumonia was probably superposed on a previously unknown multifocal lepidic adenocarcinoma, which is difficult to diagnose (31,32). Alongside the patient with lepidic adenocarcinoma, pulmonary ossification was also found in a patient with OP. Pulmonary ossification is an underrecognized pathological pattern, which has been described in several disease entities (33,34). It has previously been reported in patients with SARS-CoV-2 (35). Yamagishi et al reported on a patient with pulmonary ossification (caused by alveolar hemorrhage followed by OP) with complete regression after systemic corticosteroid therapy (36), which might be the case in our patient.

The sample adequacy was 91.2%. In their systematic review, Chami et al reported sample adequacy of transbronchial (forceps) biopsies in the setting of interstitial lung diseases (among studies with clearly defined criteria) to be 74%. In case of a SARS-CoV-2 lung infection, infected type-II alveolar cells reduce the production of pulmonary surfactant, which results in collapsed alveoli due to increased tension on their surface (37,38). Hypothetically, this could explain why we were able to obtain >20 alveoli in most of transbronchial biopsy specimens. However, this hypothesis requires further systematic exploration.

Our study has several limitations: The major limitation is its cross-sectional design. This type of study was selected to avoid repeating invasive procedures without a clear clinical indication. Second, the ongoing COVID-19 pandemic and acute COVID-19 patient overflow disabled us from systematically following these patients. Third, although cryobiopsy is superior to forceps lung biopsy (39), it was unavailable in our center. Additionally, transbronchial biopsies are prone to sampling errors (ie, divergent histopathologic diagnoses in two or more biopsy sites) and to interobserver variation between pathologists (40). This limitation was minimized by independent interpretation by two experienced pulmonary pathologists and a follow-up joint discussion. Fourth, this was a single-center study performed on a relatively small number of patients. Despite these limitations, our work offers an insight into the potential timeline of long-COVID histopathological changes.

In conclusion, our study revealed that DAD with vascular abnormalities (originally found during acute COVID-19) may persist during earlier stages of long COVID. Prolonged inflammation or early fibrosis might be two ways of DAD resolution and can be found in later stages of long COVID (after the 8th week). Vascular abnormalities associated with an inflammatory pattern (11-13 weeks) might be a natural transition from DAD to prolonged inflammation. These findings also suggest that microvascular abnormalities and long-lasting DAD might explain a slow recovery from COVID-19 pneumonia. Additionally, the persistence of vascular abnormalities up to about 12 weeks (weeks 11-13) correlates with the definition of long COVID proposed by the CDC (1,2). Further prospective studies with longitudinal follow-up at systematically defined time points are required to elucidate the significance of histopathological changes in the clinical course and prognosis of COVID-19.
